# Genetic Modification of T Cells for the Immunotherapy of Cancer

**DOI:** 10.3390/vaccines10030457

**Published:** 2022-03-16

**Authors:** Suzanne Quinn, Natasha Lenart, Victoria Dronzek, Gina M. Scurti, Nasheed M. Hossain, Michael I. Nishimura

**Affiliations:** 1 Department of Surgery, Stritch School of Medicine, Loyola University Chicago, Maywood, IL 60153, USA; nlenart@luc.edu (N.L.); vdronzek@luc.edu (V.D.); gmscurti@luc.edu (G.M.S.); mnishimura@luc.edu (M.I.N.); 2 Division of Hematology and Oncology, Stritch School of Medicine, Loyola University Chicago, Maywood, IL 60153, USA; nasheed.hossain@lumc.edu

**Keywords:** cancer immunotherapy, gene-modified TCR transduced T cells, tumor-infiltrating lymphocytes, chimeric antigen receptors, adoptive cell transfer

## Abstract

Immunotherapy is a beneficial treatment approach for multiple cancers, however, current therapies are effective only in a small subset of patients. Adoptive cell transfer (ACT) is a facet of immunotherapy where T cells targeting the tumor cells are transferred to the patient with several primary forms, utilizing unmodified or modified T cells: tumor-infiltrating lymphocytes (TIL), genetically modified T cell receptor transduced T cells, and chimeric antigen receptor (CAR) transduced T cells. Many clinical trials are underway investigating the efficacy and safety of these different subsets of ACT, as well as trials that combine one of these subsets with another type of immunotherapy. The main challenges existing with ACT are improving clinical responses and decreasing adverse events. Current research focuses on identifying novel tumor targeting T cell receptors, improving safety and efficacy, and investigating ACT in combination with other immunotherapies.

## 1. Introduction

T cells are multi-functional immune cells in the adaptive immune system that play an important role in host immunity against pathogens and cancer. Cancer presents challenges to the adaptive immune system, as tumors are derived from self-tissues and tumor antigens are often self-antigens. The immune system has developed defense mechanisms to prevent reactions against self-antigens, which may lead to immune cells often failing to recognize and destroy tumor cells. Furthermore, tumors commonly develop resistance mechanisms to evade the host immune response. As a result, new approaches have been pursued to identify immune effector mechanisms capable of recognizing tumor cells and effectively targeting tumors for destruction.

**Figure 1:**
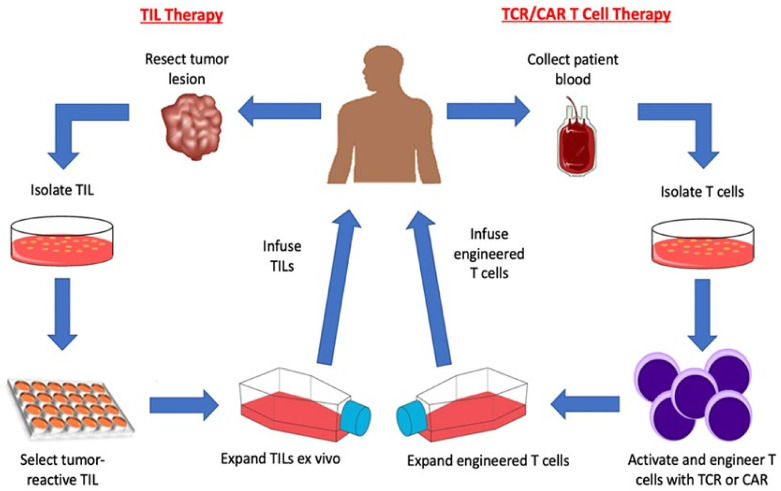
Adoptive T cell therapy strategies. Adoptive T cell therapy for treating cancer patients requires ex vivo expansion of autologous T cells for infusion back into the patient. Adoptive T cell transfer of TIL (left side of the Figure) occurs by first resecting tumor lesions from a patient and then isolating tumor-reactive T cells from that sample. The tumor-reactive T cells are then expanded ex-vivo and infused back into the patient. Adoptive T cell transfer of genetically engineered T cells (right side of the Figure) occurs by first isolating PBL-derived T cells from patient blood then genetically modifying them to express a specific TCR or CAR. The TCR or CAR engineered T cells are then expanded ex-vivo and infused back into the patient.

The use of T cells in cancer immunotherapy has been thoroughly evaluated over the last few decades. Adoptive transfer of T cells is an effective method to provide patients with a source of T cells (autologous or allogenic) capable of targeting their tumor cells (Figure 1) [1]. From lymphokine activated killer cells (LAK) to tumor infiltrating lymphocytes (TIL) to genetically engineered T cells (T cell receptor (TCR) or chimeric antigen receptor (CAR) transduced T cells), adoptive T cell transfer has shown strong potential clinically, and research has focused on identify novel T cell targets and developed corresponding receptors to improve the safety and efficacy of adoptive T cell transfer [2].

## 2. Adoptive T Cell Transfer

### 2.1. Antigen Recognition by T Cells

**Figure 2:**
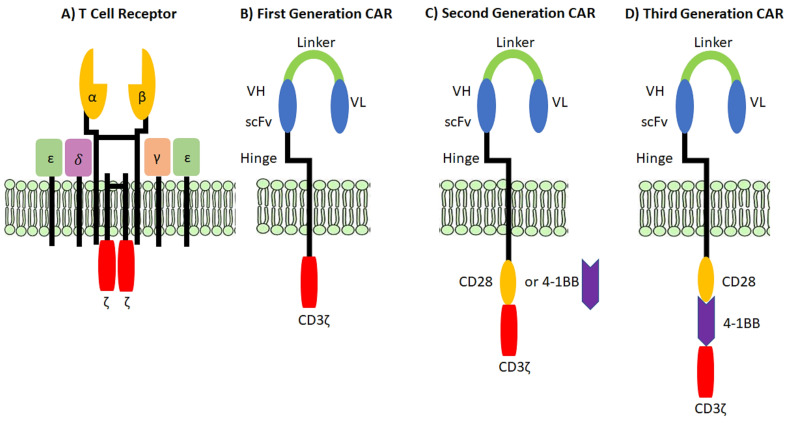
TCR structure compared to 1st, 2nd, and 3rd generation CARs. T cell receptors and chimeric antigen receptors differ significantly in their structure and how they recognize antigen. (**A**) T cell receptors are an αβ heterodimer that associates with the CD3 complex. CD3 consists of 6 chains, an ε-δ heterodimer, an ε-γ heterodimer, and a ζ-ζ homodimer. Chimeric antigen receptors consist of a scFv fused to a hinge (usually CD8), transmembrane region, and (**B**) CD3ζ (first generation CAR) or (**C**) CD28 or 4-1BB and CD3ζ (second generation CAR) or (**D**) CD28 and 4-1BB and CD3ζ (third generation CAR).

**Figure 3:**
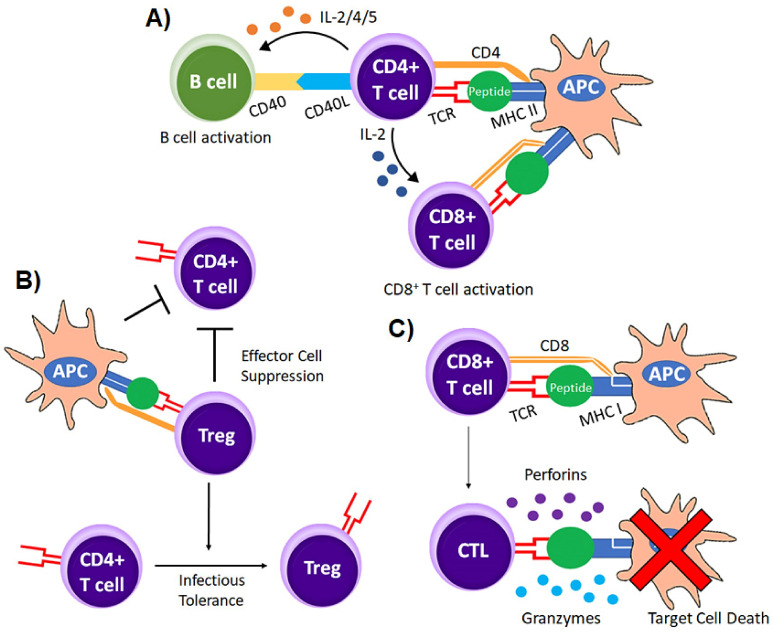
T cell subtypes. T cells are generally classified based on their cytokine production profiles and effector function. They are activated or respond to APCs or targets differently based on how antigen is presented and the other signals (cytokines, chemokines, and cell surface molecules) they receive. (**A**) CD8^+^ effector and CD4^+^ helper T cells each possess unique TCRs that interact with MHC class I or MHC class II molecules respectively on APC or target cells. In this panel, CD4^+^ T cells are providing help to CD8^+^ T cells in the form of IL-2 and other signals not shown. (**B**) CD4^+^ regulatory T cells suppress immune responses by inhibiting T activation and function. (**C**) CD8^+^ T cells are mainly effector T cells capable of inducing target cell destruction.

The key to using T cells for adoptive immunotherapy is their target specificity to target antigens of choice [1,2]. Individual T cells express unique TCRs on their cell surface, which are responsible for the antigen specificity of the T cell [3,4,5]. TCRs are αβ heterodimers that form through complex rearrangements during T cell development [4,5], and they associate with the ε, δ, γ, and ζ chains of the CD3 signaling complex (Figure 2A) [6,7]. TCR binding to the correct antigenic peptides presented on major histocompatibility complexes (MHC) on the target cell activate the T cell to kill, secrete cytokines, and/or proliferate (Figure 3) [7,8]. However, TCR binding to peptide/MHC (pMHC) is just the first step in T cell activation and function. T cells express the CD4 or CD8 coreceptors on their cell surface, which enhances the relative affinity of the TCR/pMHC and promotes signaling through the CD3 complex [9,10,11,12].

The CD4 coreceptor binds to invariant regions of MHC class II molecules while CD8 interacts with invariant regions of MHC class I molecules, making CD4^+^ T cells MHC class II restricted and CD8^+^ T cells MHC class I restricted [13,14]. The differential expression of CD4 or CD8 on T cells also tends to be a marker for their effector function. While CD8^+^ T cells can be cytolytic and have a direct effector function, CD4^+^ T cells mainly regulate immune function by releasing cytokines capable of modulating immune responses (Figure 3A). Helper T cells (T_h_) promote normal immune function whereas regulatory T cells (T_REGs_) inhibit normal T cell function (Figure 3B) [15]. CD8^+^ T cells are mainly effector cells with their cytotoxic activity directly killing virus infected cells and tumor cells (Figure 3C) [16]. Unlike most normal cells in the body, T cells can expand in vivo to fight pathogens and in vitro to large numbers, making T cells an excellent choice for adoptive cell transfer in cancer. There have been clinical trials investigating the efficacy of expanding antigen reactive T cells from patient peripheral blood samples, finding that these cells have clinical benefits, however, there are challenges in expanding antigen reactive T cells to clinically therapeutic numbers (Table 1).

### 2.2. Tumor Infiltrating Lymphocytes

The discovery and therapeutic use of TIL is an early example of adoptive T cell transfer. TIL are present in tumor lesions and are enriched for tumor-reactive T cells. Recent studies indicate that TIL not only contain T cells reactive with shared antigens, but they also contain T cells that target neoantigens [17,18,19]. TIL can function as any normal T cell by lysing tumor cells and/or secreting IL-2, IFN-γ, and other cytokines when stimulated by tumor cells [20,21]. TIL cultures are generated by first harvesting a tumor lesion, dissociating it into small fragments or a single cell suspension, culturing the cells in IL-2, and expanding them to therapeutic numbers (Figure 1) [22]. Clinical trials have determined that TIL are efficacious in mediating melanoma regression when adoptive cell transfer of TIL is combined with high dose IL-2 treatment [22,23]. Historically, TIL have been an effective therapeutic in patients with advanced malignancies, and more recently, TIL have been used to treat ovarian cancer, HPV-associated cervical cancer, renal cell carcinoma, and triple negative breast cancer (Table 1) [24,25,26] Growth of tumor-infiltrating lymphocytes from human solid cancers: summary of a 5-year experience}. TIL treatments have been demonstrated to be clinically advantageous, however, TIL therapy suffers drawbacks. Despite TIL therapy being an effective treatment, the pool of eligible patients, even for melanoma, is very limited because many tumor lesions are not easily accessible (liver, lung, brain, bone marrow, etc.) for TIL harvest. Furthermore, many TIL cultures fail to expand to therapeutic numbers, and those TIL cultures that do expand are not always tumor reactive.

Limitations with TIL led to the development of more reliable methods for generating tumor-reactive T cell cultures for use in adoptive T cell transfer protocols for cancer patients. Adoptive cell transfer of genetically modified T cells, such as TCR modified T cells or CAR T cells, is a promising method that alleviates the issues faced by TIL therapy [27,28,29].

### 2.3. Genetically Modified T Cell Receptor Transduced T Cells

Self-reactive T cells are present at low frequencies in vivo as they are usually eliminated by negative selection during T cell development [16]. Since many tumor antigens are normal proteins, the endogenous T cell repertoire usually lacks T cells with high affinity TCRs reactive with self-antigens to prevent autoimmunity. When self-reactive T cells make it through T cell development or T cells reactive with mutated self-proteins (neoantigens) are present in the periphery, they are often suppressed or exhausted in the tumor microenvironment preventing efficient tumor clearance [30,31]. Just as TIL are not functional in the tumor lesions but become therapeutic upon ex vivo activation and expansion, we first demonstrated that we could redirect the specificity of normal PBL-derived T cells with an HLA-A2 restricted, MART-1 reactive TCR (TIL 5) leading to recognition of HLA-A2^+^ MART-1^+^ tumor cells [28]. This led to the first use of TCR gene modified T cells in human beings [32]. Because of the ease of identifying TCRs that recognize melanoma antigens from TIL, most of the early trials were mainly conducted in melanoma patients [33]. Since then, adoptive transfer of TCR gene modified T cells using TCRs have been used to treat many cancer types, notably melanoma and renal cell carcinoma. More recently, clinical trials using genetically modified TCR transduced T cells targeting MAGE-A4 [34], WT-1 [35,36], NY-ESO-1 [37], HERV-E, and HPV E7, among many others, have proven that TCR transduced T cells can target nonmelanoma cancers (Table 1). While adverse events were found in some TCR gene transfer trials (to be discussed later), most trials indicated the overall approach is generally safe and well tolerated [38]. Objective clinical responses have been observed in most of these clinical trials indicating that TCR gene modified T cells have a real potential for clinical success.

**Figure 4:**
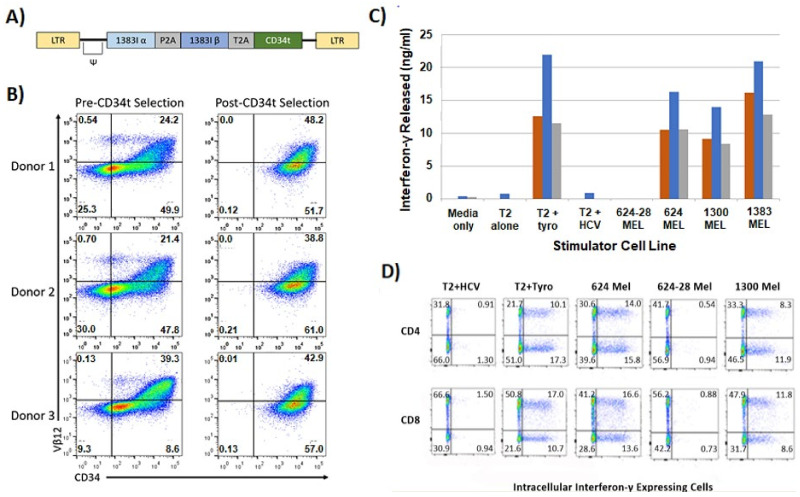
Transduction, expression, and function of TIL 1383I TCR transduced human T cells. We use retroviral and lentiviral vectors to engineer normal and cancer patient PBL-derived T cells to express TCR. (**A**) The general structure of our TIL 1383I TCR retroviral vector is shown as follows: 5′ LTR, the Ψ^+^ packaging signal, the TCR α chain fused to a P2A self-cleavage peptide fused to the TCR β chain fused to a T2A self-cleavage peptide fused to the CD34t marker gene and 3′ LTR. (**B**) Expression of the TIL 1383I TCR in PBL-derived T cells from 3 normal donors. The TIL 1383I TCR expression is based on Vβ12 expression (Y axis) and the CD34 marker gene expression (X axis). Transduction efficiency before CD34 purification (left panels) and after CD34 purification is shown (right panels). (**C**) The amount of IFN-γ released by the TIL 1383I TCR transduced T cells is shown. HLA-A2^+^ tyrosinase_(368–376)_^+^ stimulator cells include T2 loaded with 10 µg/mL tyrosinase_(368–376)_ peptide, 624 MEL, 1300 MEL, and 1383 MEL. HLA-A2^+^ tyrosinase_(368–376)_^−^ stimulator cells include T2 alone or loaded with 10 µg/mL HCV_(1406–1415)_ peptide. HLA-A2^−^ tyrosinase_(368–376)_^+^ stimulator cells were 624-28 MEL. The amount of IFN-γ released was measured in triplicate wells via ELISA. (**D**) HLA-A2 restricted, tyrosinase reactive antigen recognition by TIL 1383I TCR transduced CD8^+^ and CD4^+^ T cells was measured using intracellular IFN-γ assays. As before, HLA-A2^+^ tyrosinase_(368–376)_^+^ stimulator cells include T2 loaded with 10 µg/mL tyrosinase_(368–376)_ peptide, 624 MEL, and 1300 MEL. HLA-A2^+^ tyrosinase_(368–376)_^−^ stimulator cells include T2 cells loaded with 10 µg/mL HCV_(1406–1415)_ peptide. HLA-A2^−^ tyrosinase_(368–376)_^+^ stimulator cells were 624-28 MEL. Cells were also stained with anti-CD4, andti-CD8, and anti-CD34 (not shown) mAb. The histograms shown were gated on CD34^+^ T cells (transduced). CD4 vs. IFN-γ (top panels) and CD8 vs. IFN-γ (bottom panels) staining is shown.

There are two features of our clinical trials worth noting that were novel when the trial was initiated in 2012. First was the use of a novel high affinity HLA-A2 restricted, tyrosinase reactive TCR (TIL 1383I), which was isolated from an MHC class I restricted CD4^+^ T cell [39]. We speculated and later confirmed that tumor recognition by the TIL 1383I TCR was CD8-independent making the TIL 1383I TCR the first high affinity human TCR identified [40,41]. A retroviral vector encoding the TIL 1383I TCR (Figure 4A) was able to efficiently transduce human T cells (Figure 4B). The TIL 1383I TCR transduced T cells specifically secreted IFN-γ when stimulated with HLA-A2^+^ tyrosinase^+^ cells (Figure 4C). More importantly, both CD8^+^ and CD4^+^ T cells recognized physiologic levels of antigen presented by tumor cells meaning patients were treated with functional CD8^+^ and CD4^+^ T cells (Figure 4D) [42]. Second, a modified CD34 marker gene (CD34t) was added to the vector (Figure 4A) [43]. This CD34t cassette allowed us to enrich the transduced T cells to >99% purity using anti-CD34 immunomagnetic beads (Figure 4B) and to monitor the transduced T cells in the tissues and blood of infused patients (not shown) [42]. As of the date of this submission, we have treated 7 patients with advanced melanoma (NCT02870244, NCT01586403) and 13 patients with advanced clear cell renal cell carcinoma (NCT03354390) using CD34 enriched TCR gene modified T cells.

There are many factors that influence target recognition by TCR transduced T cells. As previously discussed, one important factor is TCR affinity [28,39,40,41,44]. We found that Jurkat cells expressing a MART-1 (TIL 5) [44], gp100 (R6C12, T4H2) [45], tyrosinase (TIL 1383I) [40], or HCV (1088, 1406) [46,47] reactive TCR secreted IL-2 when stimulated with peptide loaded T2 cells. Jurkat cells, which lack CD8 expression, only recognize the physiologic levels of antigen expressed by tumor cells if they express a CD8-independent/high affinity TCR [40,41,46,47,48]. Therefore, we concluded that our gp100 and MART-1 reactive TCRs are CD8-dependent/low affinity TCRs, whereas our tyrosinase and HCV reactive TCRs are CD8-independent/high affinity TCRs [44,45,46,47]. These results also confirmed our notion that engineering T cells with high affinity TCRs could improve the sensitivity of the T cell to antigen and generate MHC class I restricted CD4^+^ T cells [41,42,48,49]. Therefore, any TCR transduced T cell culture used for patient treatment can contain both MHC class I restricted, tumor reactive CD4^+^ and CD8^+^ T cells if engineered with a high affinity TCR (Figure 4D).

The main problem with using high affinity TCRs is they are rare in the normal T cell repertoire [39,49]. Therefore, high affinity TCRs can be produced by modifying low affinity TCRs using phage display, yeast display, HLA-A2 transgenic mice (mouse CD8 does not bind to human HLA α3 making mouse T cell CD8 independent), and by generating allospecific T cells from the peripheral blood of normal donors [12,50,51,52,53,54,55,56,57,58,59,60,61,62,63]. Since we cloned and characterized the first high affinity human TCR, TIL 1383I TCR, many groups have successfully isolated high affinity TCRs for use in TCR gene transfer.

While high affinity TCRs are effective, there are also adverse events which occur, associated with off-tumor, on-target responses, as well as off-tumor, off-target responses. For example, a TCR that targets the melanoma antigen gp100 has on-tumor/on-target activity, as well as on-target/off-tumor activity in the eye and ear resulting in vision, hearing, and balance problems [64]. Similarly, patients treated with T cells expressing a high affinity anti-CEA TCR had severe colitis [65]. Of most concern was a high affinity TCR targeting MAGE-A3 which showed efficacy as a therapy for melanoma, but was cross-reactive to MAGE-A9/12, resulting in neural toxicity [66]. A second high affinity anti-MAGE-A3 TCR was found to cross react with titin, resulting in lethal cardiac toxicity [67]. These adverse events raised legitimate concerns in the field with using high affinity TCRs, especially affinity enhanced TCRs.

Despite these adverse events, not all high affinity TCRs lead to severe adverse events. A modified high affinity TCR targeting NY-ESO-1 led to objective clinical responses and no serious adverse events [37,68]. Two patients treated with T cells transduced with our TIL 1383I TCR which had tumor regression had progressive vitiligo, but no other serious unexpected toxicities [42]. A high affinity WT1 TCR proved safe and effective in preventing relapse in AML patients [36]. These results indicate that the safety of TCR transduced T cells is a more complex problem than just TCR affinity and cross-reactivity due to affinity enhancement.

While not a new concept, the analysis of the T cell repertoire in patients treated with PD-1 blockade has thrust the concept of targeting mutated or neoantigens to the forefront [18,69,70,71]. We have known about the existence of neoantigens in mice since the earliest days of tumor immunology [72,73,74]. In early antigen cloning studies, several human neoantigens were identified but were largely ignored because of their limited clinical utility [75,76,77,78]. We also knew of the existence of tumor-specific T cells in humans because they recognize only the autologous tumor [79,80,81]. Adverse events observed in some TCR gene transfer clinical trials, combined with the fact that neoantigens are not expressed on normal tissues, have led some in the field to develop strategies to identify and clone TCRs that target neoantigens [70,82,83,84,85,86,87,88,89]. However, some of the most exciting TCRs target shared neoantigens such as mutant TGFβRII [89], KRAS [90,91], and TP53 [92,93]. As the technology improves, the feasibility of targeting neoantigens with TCR gene modified T cells will improve, adding a whole new treatment option for patients with advanced cancer.

Despite the excitement in the TCR gene transfer field, there are limitations that detract from using TCRs for adoptive T cell therapy. One main hurdle is the limitations that MHC restriction place on patient eligibility [2,94]. Another hurdle is many tumors exhibit MHC and/or antigen processing loss, reducing the ability of a T cell to recognize the tumor [95]. Another class of genetically modified T cells, called chimeric antigen receptor (CAR) T cells, do not depend on costimulation or cytokine signaling to activate because of their unique structure. As a result, CAR T cell activation after tumor cell recognition is more sensitive compared to TCR transduced T cells.

### 2.4. Chimeric Antigen Receptor (CAR) T cells

CARs are artificially generated receptors that have been built to specifically target antigens expressed on the cell surface [27]. T cells are typically engineered to express CARs by transducing patient T cells with virus that encodes the DNA construct. The resulting CAR T cells are then expanded ex vivo and infused back into the patient (Figure 1). Although CAR and TCR transduced T cells are typically produced for patient treatment using similar methods, there are significant structural and operational differences between the two cell types [2,96].

There are two primary distinctions between TCRs and CARs that lead to major differences in their function. Firstly, TCRs target peptide molecules that are bound to MHC molecules expressed on the surface of cells, while CARs target cell surface molecules independent of MHC binding [2,96]. Secondly, CARs possess all of the molecules required for antigen binding and T cell activation, whereas TCRs are only able to bind to MHC molecules to relay the first signal of T cell activation, meaning that secondary and tertiary signaling is required for T cell activation after the TCR initially binds to antigen [2,96]. The structural differences between a TCR and various generations of CAR molecules are shown in Figure 2. Although different generations of CARs vary slightly (Figure 2B), there are a few fundamental structures that all CAR molecules possess.

CARs are considered chimeric because they are constructed from molecules that provide various levels of functionality to the receptor. The most basic CAR, known as a first-generation CAR (Figure 2B), is made up of a single chain variable fragment (scFv) that contains the heavy and light chain antigen binding regions isolated from an immunoglobulin molecule that is fused to a CD3ζ signaling chain via a hinge and transmembrane domain (Figure 3) [97,98,99,100]. Early CAR molecules used Fc receptor γ (FcRγ) signaling domains, rather than CD3ζ [101]. However, FcRγ domains contain only one immunoreceptor tyrosine-based activation motif (ITAM), and ITAM signaling is necessary for the activation and function of T cells [2,102]. On the other hand, CD3ζ domains contain three ITAMs, which leads to more effective T cell signaling and activation [101]. Therefore, the use of FcRγ domains in CAR constructs has been phased out in favor of CD3ζ signaling chains [101]. Although first generation CARs contain both an antigen binding region as well as a T cell activation signaling domain, they lack a costimulatory signaling domain [99]. Even though T cells can activate without costimulatory signaling, costimulation by molecules like CD28 or 4-1BB are known to drive optimal T cell activation, leading to increased persistence and development of long-term memory [12,103,104,105,106]. Many CAR configurations have been evaluated including using different costimulatory cassettes and/or altering the number and position of the costimulatory cassettes [107,108]. As a result, first generation CARs have since been modified to include costimulatory cassettes to improve the functionality of CAR T cells in vivo [103,104,109,110,111]. The most common costimulatory cassettes included in CAR constructs are CD28, 4-1BB, ICOS, or OX40 (Figure 2C) [100,104,109,110,111,112,113,114,115,116]. Third generation CARs include two distinct costimulatory cassettes, such as 4-1BB and CD28 together (Figure 2D) [117,118,119,120]. Both second and third generation CAR T cells demonstrate enhanced proliferation, increased cytotoxic activity, and sustained anti-tumor effects compared to first generation CARs [120,121,122,123]. Although third generation CAR T cells may exhibit increased potency, concerns have arisen regarding their use because serious adverse events have been recorded after their infusion, likely due to reduced activation thresholds that lead to signaling leakage and T cell dysfunction [121,124,125]. As a result, clinical research has been primarily focused on developing new targets for and enhancing the anti-tumor efficacy of second generation CARs, leading to the development of fourth generation CARs [121,122,123,124].

**Figure 5:**
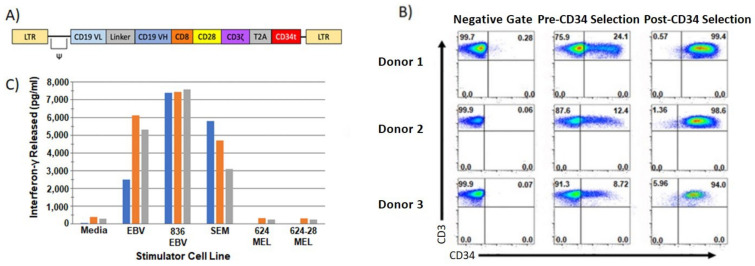
Transduction, expression, and function of CD19 transduced human T cells. We use retroviral and lentiviral vectors to engineer normal and cancer patient PBL-derived T cells to express TCR. (**A**) The general structure of our CD19 CAR retroviral vector is shown as follows: 5′ LTR, the Ψ^+^ packaging signal, the CD19 CAR which consists of CD19 V_L_ fused to CD19 V_h_ by a flexible linker followed by CD8 hinge then a CD28 cassette followed by CD3ζ. The CAR is fused to a T2A self-cleavage peptide fused to the CD34t marker gene followed by the 3′ LTR. (**B**) Expression of the CD19 CAR in PBL-derived T cells from 3 normal donors. CD19 CAR expression is based on the CD34 marker gene expression. Transduction efficiency of untransduced (Negative Gate), pre-CD34 selection, and post-CD34 selection is shown. Histograms represent CD3 expression (Y axis) and CD34 expression (X axis). (**C**) The amount of IFN-γ released by the CD19 CAR transduced T cells is shown. CD19^+^ stimulators include the line EBV, 836 EBV, and SEM and CD19^−^ stimulators include 624 MEL and 624-28 MEL. The amount of IFN-γ released was measured in triplicate wells via ELISA.

CAR T cells have been utilized extensively in clinical trials for the treatment of cancer (Table 1). This form of therapy has been most successful in treating hematological malignancies, especially B cell leukemias and lymphomas [124,125,126,127,128,129]. CAR constructs that target CD19 on B cells are extremely effective, and multiple reports have demonstrated that anti-CD19 CAR T cells produce consistent anti-tumor effects in patients [130,131]. In one study, 2 out of 3 chronic lymphocytic leukemia patients that received CD19 CAR T cells displayed complete responses to treatment [124]. Clinical trials reported from other institutions have observed similar results, and data suggests that overall about 25% of patients demonstrate complete responses [130,132,133,134]. The CD19 CAR construct used in our clinical trial (NCT04214886) contains a CD28 cassette and our standard CD34t cassette for purification of the CAR transduced T cells (Figure 5A). Following purification, the cultures are ≥94% pure CD19 CAR T cells (Figure 5B) and they secrete large amounts of IFN-γ when stimulated with CD19^+^ tumors, but not CD19^−^ tumors (Figure 5C). We found that second generation CAR constructs are quite effective at generating tumor reactive T cell cultures. We also found that a lower number of CAR transduced T cells are needed (generally less than 1 × 10^8^ transduced T cells) to achieve objective clinical responses than TCR transduced T cells (unpublished). These promising results have led to FDA approval of five CAR T cell products: an anti-CD19 CAR with a 4-1BB costimulatory cassette called tisagenlecleucel (Kymirah-Novartis)**,** an anti-CD19 CAR with a CD28 costimulatory cassette called axicabtagene (Yescarta-Kite/Gilead), an anti-BCMA CAR with a 4-1BB costimulatory cassette called idecabtagene (ABECMA-Celgene/BMS), an anti-CD19 CAR with a 4-1BB costimulatory cassette called lisocabtagene (Breyanzi-Juno/BMS), and an anti-CD19 CAR with a CD28 costimulatory cassette called brexucabtagene (Tecartes-Kite/Gilead) [135,136,137,138,139,140,141,142,143]. The encouraging results obtained with CAR T cells targeting B cell malignancies have not been recapitulated in studies targeting solid tumors [144]. A number of clinical trials have been conducted to test CAR T cells against solid tumors. CARs against IL13Rα2, HER2, MUC1, and others have all been used as targets against solid tumors in clinical trials for patients with gliomas, advanced sarcomas, pancreatic cancer, renal cell carcinoma, mesothelioma, and other tumors [116,144,145,146,147,148,149,150,151,152,153,154,155,156,157,158,159,160,161]. However, CARs targeting solid tumors have not achieved the same level of clinical success as anti-CD19 CAR T cells [162]. The biological differences between hematologic malignancies and solid tumors, such as solid tumor density, solid tumor heterogeneity, and hostile solid tumor microenvironments are likely part of the reason why CAR T cells struggle to eradicate solid tumors [144,162]. Another concern is that serious adverse events have occurred in early CAR trials in patients with solid tumors [125,162,163]. These toxicities are often reversible or manageable and new insights into CAR T cell mechanistic interactions have allowed researchers to reduce the probability of toxicity after infusion [2,161,162,163].

Cancer immunotherapies, including TILs, TCR modified T cells, and CAR T cells, have proved to be potentially life-saving forms of therapy. Despite their success, there are still a number of challenges that prevent these therapies from achieving their maximum potential. Hostile tumor microenvironments, antigen escape, and tumor heterogeneity can inhibit proper engraftment and long-term function of engineered T cells [100]. As a result, new methods of treatment have been sought out to enhance anti-tumor effects of adoptive T cell therapy to improve the frequency of clinical success.

## 3. Summary

The use of gene modified T cells for cancer immunotherapy has become increasingly popular. Adoptive T cell therapy focuses heavily on genetically modifying autologous T cells isolated from patients. This form of therapy is particularly effective because patients receive tumor-reactive T cells that can efficiently recognize and target tumor cells, which endogenous T cells are typically not able to do very well. Stimulating immune cells within the tumor microenvironment is critical to promoting T cell-mediated tumor regression. Despite the challenges these therapies currently face, combining adoptive T cell therapy with other treatment methods to stimulate T cell function poses a potential solution to overcome those hurdles and improve clinical response rates. Research is currently focused on developing novel tumor targets and testing these therapies in the clinic. As clinical response rates improve and new treatments become commercially available, the accessibility and popularity of these therapies will increase as well.

## Figures and Tables

**Table 1:** Selected T cell immunotherapy clinical trials.

Clinical Trial Number and Title	Status	Phase	T-Cell Source	Location
NCT00338377: Lymphodepletion Plus Adoptive Cell Transfer with or without Dendritic Cell Immunization in Patients with Metastatic Melanoma	Not recruiting	II	TIL	Texas, United States
NCT00604136: Treatment of Metastatic Melanoma with Tumor Infiltrating Lymphocytes and IL-2 Following Lympho-Depleting Chemotherapy	Unknown	II	TIL	Israel
NCT01740557: Genetically Modified Therapeutic Autologous Lymphocytes Followed by Aldesleukin in Treating Patients with Stage III or Metastatic Melanoma	Not recruiting	I-II	Nerve Growth Factor Receptor and CXCR2 Transduced TIL	Texas, United States
NCT01883323: Tumor-Infiltrating Lymphocytes and Low-Dose Interleukin-2 Therapy Following Cyclophosphamide and Fludarabine in Patients with Melanoma	Completed	II	TIL	Ontario, Canada
NCT01946373: T Cell Transfer with or without Dendritic Cell Vaccination in Patients with Melanoma	Recruiting	I	TIL	Sweden
NCT02278887: Study Comparing TIL to Standard Ipilimumab in Patients with Metastatic Melanoma (TIL)	Recruiting	III	TIL	Denmark and Netherlands
NCT02354690: Vemurafenib and TIL Therapy for Metastatic Melanoma	Completed	I/II	TIL	Denmark
NCT02379195: Peginterferon and TIL Therapy for Metastatic Melanoma	Completed	I/II	TIL	Denmark
NCT02424916: Adoptive Transfer of Specific Melanoma Antigens CD8+ T Cells in Metastatic Melanoma Patients	Completed	I/II	Melan-A and MELO-1 Antigen Specific T Cells	France
NCT02959905: Treatment of Advanced Solid Tumor with TSA-CTL	Unknown	I	Tumor-Specific Antigen (TSA) Induced Cytotoxic T Lymphocytes	China
NCT02568748: Evaluation of Cytokine-induced Killer (CIK) Cells as Therapy or Adjuvant Treatment for Advanced HCC	Unknown	III	Cytokine-Induced Killer Cells	Egypt
NCT02498756: Cytokine-Induced Killer Study for Patients with Stage II Melanoma	Not yet recruiting	II	Cytokine-Induced Killer Cells	China
NCT00779337: Epstein-Barr Virus (EBV)-Specific T Cells as Therapy for Relapsed/Refractory EBV-Positive Lymphomas (EPL)	Completed	I	EBV-Specific Cytotoxic T Lymphocytes	Australia
NCT02408016: Genetically Modified T Cells in Treating Patients with Stage III-IV Non-Small Cell Lung Cancer or Mesothelioma	Terminated	I/II	WT-1 TCR Transduced PBL T Cells	Washington, United States
NCT02457650: T Cell Receptor-Transduced T Cells Targeting NY-ESO-1 for Treatment of Patients With NY-ESO-1- Expressing Malignancies	Unknown	I	NY-ESO-1 Specific TCR Transduced PBL T Cells	China
NCT02770820: Laboratory-Treated (Central Memory/Naive) CD8+ T Cells in Treating Patients with Newly Diagnosed or Relapsed Acute Myeloid Leukemia	Terminated	I/II	WT-1 TCR Transduced PBL T Cells	Washington, United States
NCT02774291: Anti-ESO mTCR-transduced Autologous Peripheral Blood Lymphocytes and Combination Chemotherapy in Treating Patients with Metastatic Cancer	Unknown	I	NY-ESO-1 Specific Murine TCR Transduced PBL T Cells	New York, United States
NCT02858310: E7 TCR T Cells for Human Papillomavirus-Associated Cancers	Recruiting	I/II	E7 Specific TCR Transduced PBL T Cells	Maryland, United States
NCT03354390: HERV-E TCR Transduced Autologous T Cells in People with Metastatic Clear Cell Renal Cell Carcinoma	Recruiting	I	HERV-E Specific TCR Transduced PBL T Cells	Maryland, United States
NCT00910650: Study of Gene Modified Immune Cells in Patients with Advanced Melanoma (F5)	Completed	II	MART-1 F5 TCR-Transduced PBL T Cells	California, United States
NCT01967823: T Cell Receptor Immunotherapy Targeting NY-ESO-1 for Patients with NY-ESO-1 Expressing Cancer	Completed	II	NY-ESO-1 Specific TCR Transduced PBL T Cells	Maryland, United States
NCT02096614: Investigator Initiated Phase 1 Study of TBI-1201	Completed	I	MAGE A4-Specific TCR Transduced PBL T Cells	Japan
NCT02111850: T Cell Receptor Immunotherapy Targeting MAGE-A3 for Patients with Metastatic Cancer Who Are HLA-DP0401 Positive	Completed	I/II	MAGE A3-Specific TCR Transduced PBL T Cells	Maryland, United States
NCT02830724: Administering Peripheral Blood Lymphocytes Transduced with a CD70-Binding Chimeric Antigen Receptor to People with CD70 Expressing Cancers	Recruiting	I/II	CD70-Specific CAR Transduced PBL T Cells	Maryland, United States
NCT03851146: A Study of Anti-Lewis Y Chimeric Antigen Receptor-T Cells (LeY-CAR-T) in Patients with Solid Tumours (LeY-CAR-T)	Not yet recruiting	I	Lewis Y-Specific CAR Transduced PBL T Cells	Australia
NCT05063682: The Efficacy and Safety of Brain-Targeting Immune Cells (EGFRvIII-CAR T Cells) in Treating Patients with Leptomeningeal Disease From Glioblastoma. Administering Patients EGFRvIII -CAR T Cells May Help to Recognize and Destroy Brain Tumor Cells in Patients (CARTREMENDOUS)	Not yet recruiting	I	EGFRvIII-Specific 4-1BB CAR Transduced PBL T Cells	Finland and India
NCT04206943: Study of CD19 Specific Chimeric Antigen Receptor Positive T Cells (CAR-T) in ALL and NHL (ISIKOK-19)	Unknown	I/II	CD19-Specific CAR Transduced PBL T Cells	Turkey
NCT03937544: Intravenous Autologous CD19 CAR-T Cells for R/R B-ALL	Recruiting	II/III	CD19-Specific CAR Transduced PBL T Cells	Malaysia
NCT02482532: Vaccine Enriched, Autologous, Activated T-Cells Directed to Tumor in Patients with Relapsed/Refractory Melanoma	Completed	I	GD2-CAR Transduced PBL T Cells	Kansas, United States

TIL, tumor-infiltrating lymphocytes; IL, interleukin; EBV, Epstein-Barr virus; TCR, T cell receptor; PBL, peripheral blood lymphocytes; HERV, human endogenous retrovirus; CAR, chimeric antigen receptor; EGFR, epidermal growth factor receptor; ALL, acute lymphoblastic leukemia; NHL, non-Hodgkin’s lymphoma; R/R, relapsed or refractory. Trial identification and information compiled from ClinicalTrials.gov (accessed 3 March 2022).

## Data Availability

The data presented in this review is available in this article or upon request. Clinical trial information is publicly assessable on clinicaltrials.gov.
